# A dataset of text prompts, videos and video quality metrics from generative text-to-video AI models

**DOI:** 10.1016/j.dib.2024.110514

**Published:** 2024-05-11

**Authors:** Iya Chivileva, Philip Lynch, Tomás E. Ward, Alan F. Smeaton

**Affiliations:** a School of Computing, Dublin City University, Glasnevin, Dublin 9, Ireland; b Insight SFI Research Centre for Data Analytics, Dublin City University, Glasnevin, Dublin 9, Ireland

**Keywords:** Generative AI, Video annotation, Video naturalness, Video perception, Video alignment

## Abstract

Evaluating the quality of videos which have been automatically generated from text-to-video (T2V) models is important if the models are to produce plausible outputs that convince a viewer of their authenticity. This paper presents a dataset of 201 text prompts used to automatically generate 1,005 videos using 5 very recent T2V models namely Tune-a-Video, VideoFusion, Text-To-Video Synthesis, Text2Video-Zero and Aphantasia. The prompts are divided into short, medium and longer lengths. We also include the results of some commonly used metrics used to automatically evaluate the quality of those generated videos. These include each video's naturalness, the text similarity between the original prompt and an automatically generated text caption for the video, and the inception score which measures how realistic is each generated video.

Each of the 1,005 generated videos was manually rated by 24 different annotators for alignment between the videos and their original prompts, as well as for the perception and overall quality of the video. The data also includes the Mean Opinion Scores (MOS) for alignment between the generated videos and the original prompts.

The dataset of T2V prompts, videos and assessments can be reused by those building or refining text-to-video generation models to compare the accuracy, quality and naturalness of their new models against existing ones.

Specifications Table

**Table:** 

Subject	Computer Vision and Pattern Recognition
Specific subject area	Dataset for the evaluation of text-to-video generation models which automatically generate videos from text prompts.
Data format	Raw videos as .mp4 and .gif; text prompts as .txt; video metric scores and annotations as .csv.
Type of data	Text prompts in .txt file, videos in .mp4 and in .gif formats, .CSV file of scores for video naturalness, automatic measures of similarity between the prompts and videos and summary scores from human annotations of video quality and alignment between prompts and videos.
Data collection	Text prompts were selected by combining content generated by ChatGPT with manual curation. Video data was created by using the text prompts as inputs to 5 popular text-to-video generation models. The resulting videos were analysed using metrics for their quality and for the naturalness as well as their similarity to the prompts used to generate them. Human annotators then rated the videos for quality and for closeness to their original prompts, using an online annotation tool.
Data source location	Annotators who performed manual video annotations online were based in the area around Dublin, Ireland. All other data was generated from computer processing.
Data accessibility	Repository name: Figshare Data identification number: 10.6084/m9.figshare.24078045.v3 Direct URL to data: https://doi.org/10.6084/m9.figshare.24078045.v3 GitHub URL for code for implementing video naturalness calculation: https://github.com/Chiviya01/Evaluating-Text-to-Video-Models Github URL for statistical data extracted from videos used to train the naturalness classifier: https://github.com/Chiviya01/Evaluating-Text-to-Video-Models/blob/main/Video_Naturalness/Classifier/Statistical_Video_Data.csv

## 1 Value of the Data

• This first-of-its-kind dataset can be reused by researchers working on text-to-video generation models who wish to evaluate the accuracy and naturalness of their own generated videos. Researchers can re-use the prompts from this data to generate their own videos and compute results from automatic metrics for naturalness, quality and alignment with the text prompt as well as re-using the code provided to calculate video naturalness, and then compare those results with the metric results from videos in this dataset.
• The dataset may be used to train text-to-video and/or video-to-text deep learning models.
• The text prompts vary in length with 87 of them being short (4 to 8 words), 43 are of average length (9 to 13 words) and 71 are longer than 13 words and this allows researchers to explore the relationship between prompt length and the quality of a generated video.
• The dataset gives insights into the comparative performances of 5 popular text-to-video models, namely Tune-a-Video, VideoFusion, Text-To-Video Synthesis, Text2Video-Zero and Aphantasia.
• The dataset is freely available for public download.

## 2 Background

In the field of AI-generated images, recent work [1] has compared the quality of images generated from a collection of text-to-image (T2I) models. This used automatically-computed metrics for image quality as well as human evaluations of perception and alignment of the image to the text prompt used to generate it. That work also included the release of a database of images and the prompts and model parameters used to generate them as well as human evaluations and outputs from automatic metrics.

The creation and release of the dataset described here follows a similar sequence to [1] except we address evaluating the quality of text-to-video (T2V) instead of text-to-image generative models. The dataset includes the videos generated from each of 5 popular T2V models using the same text prompts, as well as metric values for video quality, naturalness, perception and alignment. It also includes human annotations of those generated videos which also measure video quality and alignment between the text prompt and the generated video.

The creation of the dataset allows researchers to directly compare the performance of their own text-to-video (T2V) models against others from the literature, using a common dataset, common automatically-computed metrics and human annotations.

## 3 Data Description

**Fig. 1:**
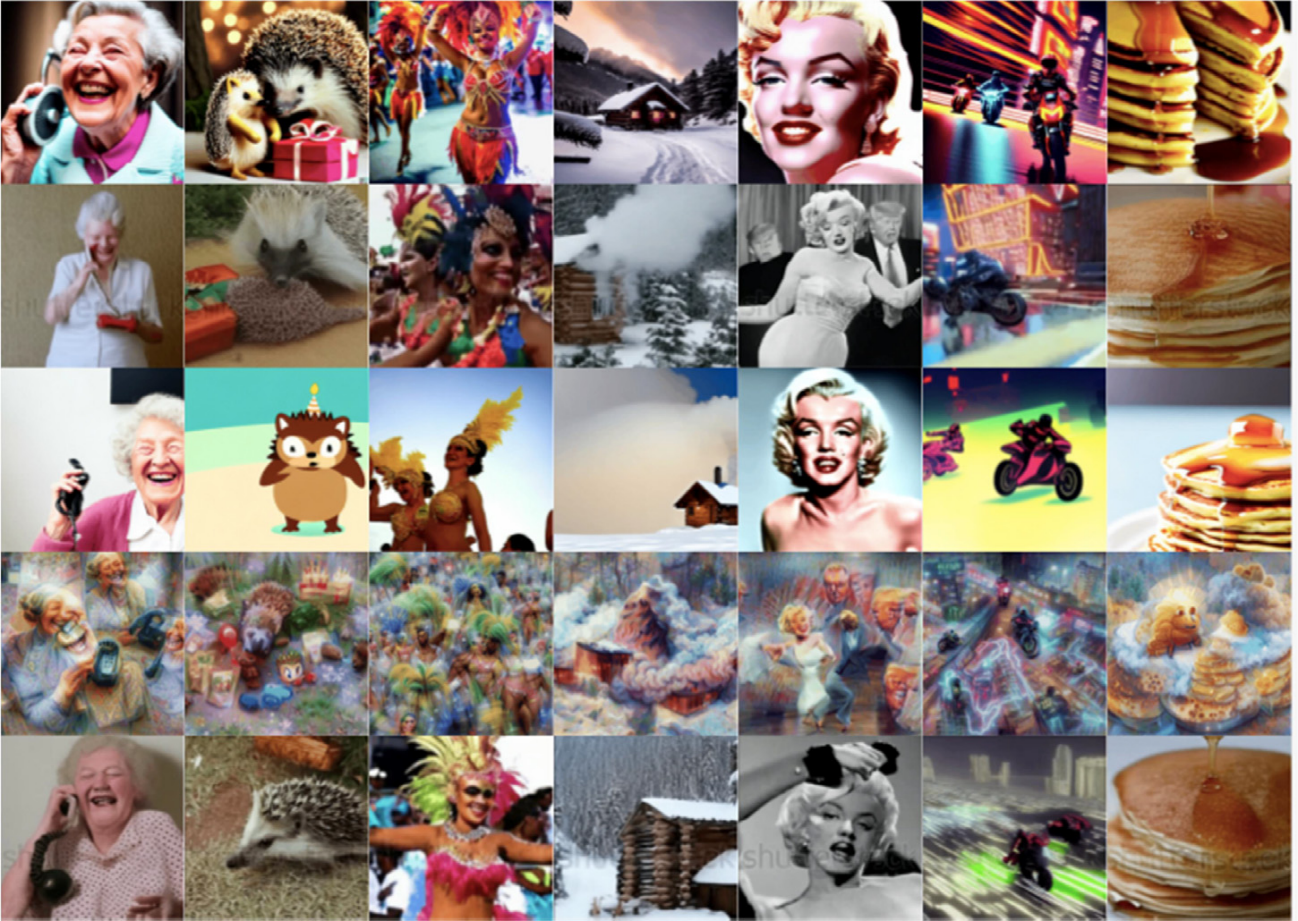
Samples from our generated videos – rows show frames generated by Text2Video-Zero, Text-to-Video Synthesis, Tune-a-Video, Aphantasia and Video Fusion respectively while the columns are frames from the same text prompts.

The dataset comprises 201 text prompts which were used in 5 T2V models to generate 1005 T2V model videos. We carefully selected the 201 prompts by combining content generated by ChatGPT with manual curation. The compilation covers a broad range of topics including influential figures, notable places, and cultural events like Easter and the Brazilian Carnival. 87 of the prompts are short (4 to 8 words), 43 are of average length (9 to 13 words) and 71 are longer than 13 words. The collection of prompts offers a diverse range of videos, spanning from practical scenarios to creative concepts. The videos encompass a variety of actions, relationships, and visual styles. Sample frames from the collection of generated videos are shown in Fig. 1.

The text prompts are in the file “text_prompts_for_video_generation.txt” with one prompt per line, each ended with a full stop.

The videos generated from each of 5 T2V models are in MP4 format except for those generated from Tune-a-Video which are animated GIFs. The 1005 videos are grouped into 5 Zipped folders, one for each model, and the expanded Zipfiles are named Aphantasia, Tune-a-video, Video_Fusion, ModelZero (for Text2Video-Zero) and T2VSynthesis respectively.

The final file in the dataset is “T2V Numerical Data.csv” which contains a row entry for each of the 1005 videos with a column for each of the “unique_video_name”, the “original prompts”, the “model” used to generate the video (one of Aphantasia, ModelZero, T2VSynthesis, Video_Fusion or Tune-a-Video), the “prompt length” (one of long, short or average), the “naturalness_score” for the video (in the range 0 to 1.0), a “text_similarity” score (in the range 0 to 1.0) between the originating prompt and an automatically-determined caption for the generated video which is a similarity algorithm described later, the “BLIP_SIM” similarity (in the range 0 to 1.0) between the originating prompt and the automatically-determined caption, the “inception score” and the mean opinion score (MOS) from the anonymized 24 manual annotations of the video for both alignment between the originating prompt and the generated caption (“MOS Alignment”), and for the perceptual quality of the generated video (“MOS Perception”). The final column contains the average of the two MOS values.

## 4 Experimental Design, Materials and Methods

In 2022 the first open-source T2V model called **Tune-a-Video** was released by Wu et al. [2] introducing a mechanism that uses the Stable Diffusion model [3] for video generation. This model is built on state-of-the-art T2I diffusion models and involves a tailored spatio-temporal attention mechanism and an efficient one-shot tuning strategy. It served as an inspiration for the rapid development of other open-source models including **VideoFusion** [4] which, in 2023, uses a decomposed diffusion process to resolve per-frame noise as a base noise that is shared among all frames leading to smoother video output. **Text-to-Video Synthesis**, also based on the work described in [4] in 2023 is also a multi-stage text-to-video generation diffusion model which consists of text feature extraction, a text feature-to-video latent space diffusion model, and video latent space to video visual space. **Text2Video-Zero** [5] in 2023 takes a low-cost zero-shot text-to-video generation approach, leveraging the power of Stable Diffusion and tailoring it for video. Finally **Aphantasia** [6] also from 2023, is a collection of text-to-image tools, evolved from the artwork of the same name which also generates video format outputs.

Having generated a total of 1005 videos from the set of 201 prompts we then calculated the values of a number of automatic and manual metrics to enhance the usefulness of the dataset.

One of the most commonly used automatic metrics for evaluating video quality is Inception Score (IS) [7] which was developed as an alternative to human evaluation and aims to measure both image quality and diversity. It relies on the ``inception network" [8] to generate a class probability distribution for images and IS scores for teach video are included in this dataset.

Image naturalness refers to how realistic and free of distortions or artefacts an image appears. Naturalness is related to quality, which encompasses aspects such as sharpness, contrast, and colour accuracy, but naturalness specifically focuses on the realism of an image.

We developed and applied a classifier for video naturalness for which we collected several statistical measures from each video, including:

• Texture score measures the degree of uniformity in a video frame's texture since natural images, such as landscapes or animal fur, tend to have more complex textures than synthetic images. After converting to grayscale and applying a Gaussian blur to reduce noise we apply Sobel edge detection in the x and y directions and calculate the magnitude of the gradient. The variance of this magnitude is the texture score.
• The sharpness score measures the amount of high-frequency content in a video frame, indicative of the image's level of detail. It is calculated by applying a sharpening filter to the image and then taking the RMS difference between the original and the filtered image.
• The colour distribution score is a measure of the uniformity of colour in a frame, exploiting the characteristic of a uniform or artificial colour distribution in a non-natural image. It is calculated by applying K-means clustering with *K* = 2 to the A and B channels of the frame's LAB representation. This score is the proportion of pixels in the cluster with the lowest A channel value.
• The spectral score measures the extent to which a frame differs from the natural image statistics in the Fourier domain. The function calculates the mean and standard deviation of each colour channel and computes the spectral score as the sum of standard deviations divided by the sum of means.
• The entropy score uses the Shannon entropy formula [9] which measures the level of randomness or disorder in pixel values. Natural images tend to have a higher degree of order and lower entropy than non-natural ones.
• The contrast score measures differences between the lightest and darkest parts of a video frame by dividing the standard deviation of pixel intensities by the mean intensity.
• Oriented FAST and Rotated BRIEF (ORB) is a feature detection algorithm [10] to compute statistics about the key points in a frame including the mean and standard deviations of the distances between key points and of the lengths of the descriptors associated with those key points.
• The number and sizes of blobs is detected using the Laplacian of Gaussian (LoG) method [11]. Blobs are regions in a video frame with a relatively uniform intensity that stand out compared to the surrounding area.
• The Naturalness Image Quality Evaluator (NIQE) is a no-reference image quality assessment metric [12] based on the observation that natural images tend to exhibit a unit-normal Gaussian characteristic in their luminance values. NIQE uses a set of natural scene statistics (NSS) that captures the statistical regularities present in natural scenes that are not present in unnatural or distorted images.

To enable processing, a YUV444 video frame is reshaped from planar to interleaved format, which represents colour information in terms of brightness (Y) and colour (U and V), with 8 bits allocated to each channel. NIQE scores were calculated for the grayscale frames and for the Y, U and V channels in the YUV444 video frames separately as this provides a better visual representation of the image [13].

To train the classifier for video naturalness we also calculated a Modified Inception Score (MIS) for each video which operates on a similar principle to Inception Score mentioned earlier by calculating the mean probability distributions of all frames in a generated video. We modified the IS metric to return a larger value if the mean probability distribution in a video has low entropy. Essentially, if the Inception model assigns a greater probability to one particular class throughout the frames in a video, MIS will produce a larger value. We achieved this by setting the marginal distribution to the uniform distribution.

We collected all video feature data described above from 187 videos comprising 92 natural and 95 non-natural scenes. The 187 videos used to train the naturalness classifier are a mixture of realistic-looking and synthetic video of natural scenes. A set of 21 low level features and quality metric values from these videos were used to train the classifier, including Niqe, Brisque, Sharpness, Entropy, Contrast, the number, size and distribution of blobs and inception scores. These low-level feature values are available on a public Githib with the URL given earlier. We approached the naturalness classifier task as a binary classification problem and manually assigned each video a label indicating natural or not. We trained three classifiers, AdaBoost, a Bagging classifier with a DecisionTree base and XGBoost. To optimise the performance of each classifier, we employed GridSearch. We evaluated the classifiers' performance using F1 on training, validation, and test sets. The XGBoost classifier performed the best on unseen data and was used to calculate the values of naturalness score for each video in the dataset.

To measure alignment between the original text prompt and the generated video we measured the semantic similarity between captions for the generated videos and the original text prompts. The process involves generating captions for each video frame using the BLIP-2 [14] image caption generator. In our approach to measuring alignment for generated videos we combine BERT and Cosine similarities.

BERT (Bidirectional Encoder Representations from Transformers) models [15] measure the similarity between two pieces of text. BERT is designed to capture more nuanced and complex semantic relationships between sentences or between captions and prompts in this case, whereas Cosine similarities only consider surface-level similarity based on word overlap. Our similarity metric penalises the BERT similarity score with the Cosine similarity score ensuring that the combined similarity shown in Eq. (1) reflects both surface-level and deeper semantic similarities between captions and prompts. After multiple experiments we determined the optimal ratio between BERT and Cosine similarities to be 0.75:0.25.

(1) $$\begin{array}{c} Text\;Similarity=\left\{ \begin{array}{cc} 0.25\;\left(Cos\;sim\right)\;+\;0.75\;\left(BERT\;sim\right), & \;if\;Cos\;sim\neq 0\\ 0.5\;\left(BERT\;Sim\right) & \;otherwise \end{array}\right. \end{array}$$

**Fig. 2:**
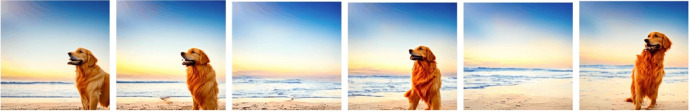
Selected frames from a generated video with the prompt “A golden retriever eating ice cream on a beautiful tropical beach at sunset''. Note that 2 of the frames are missing the dog.

Given that some frames in generated videos may exhibit significant distortions or omissions or not contain recognisable objects such as in Fig. 2 where two frames in a generated video do not include a dog, we calculate the weighted textual similarity for a generated video of *n* frames as $\frac{1}{n}\sum_{i=1}^{n}w_{i\cdot }sim_{i}$. The weights are assigned based on the frequency of each caption in the overall list of generated captions and these are the values that appear in the “text_similarity” column in the dataset.

To obtain human quality evaluation scores for the generated videos we recruited volunteers to rate videos remotely and in their own time with each person given 10 days to complete the task across up to 10 interactive sessions. The annotators rated each video on a scale of 1 (low) to 10 (high) for two aspects, alignment and perception. They were asked to rate each video on a scale of 1 to 10 for each of two categories and were given the following definitions of alignment and of perception:

• “Alignment Score reflects the compatibility between the generated video and the text of the original prompt so consider all elements in the text as crucial.”
• “Perception Score rates the perceptual quality of the video taking into account issues such as visual clarity: How clear and sharp are the visuals in the video clip? Are there any visual artifacts, blurriness, or pixelation? How accurately do the colours represent the real-world scene or intended visual style as some of these are real world, others cartoon, others impressionist, etc.? Are there any colour distortions or inaccuracies? What is your overall impression of the quality of the video produced?”

24 (16 male, 8 female) adult annotators, mostly graduate students, completed two ratings of each video giving 1005 videos x 24 annotators x 2 ratings = 48,240 quality ratings. Annotators were rewarded with a gift token when they completed annotating the videos.

In assessing the quality of still images the ``de facto'' metric is mean opinion score (MOS) [16] which is the mean of the opinions and ratings of human evaluators gathered according to some numeric or qualitative scale such as we use here. In [17] the authors proposed that the standard deviation of opinion scores reflects the subjective diversity while more recently [18] proposed that as well as the mean of the opinion scores, researchers should assess quality in terms of the distribution of opinion scores, not just the standard deviation.

**Fig. 3:**
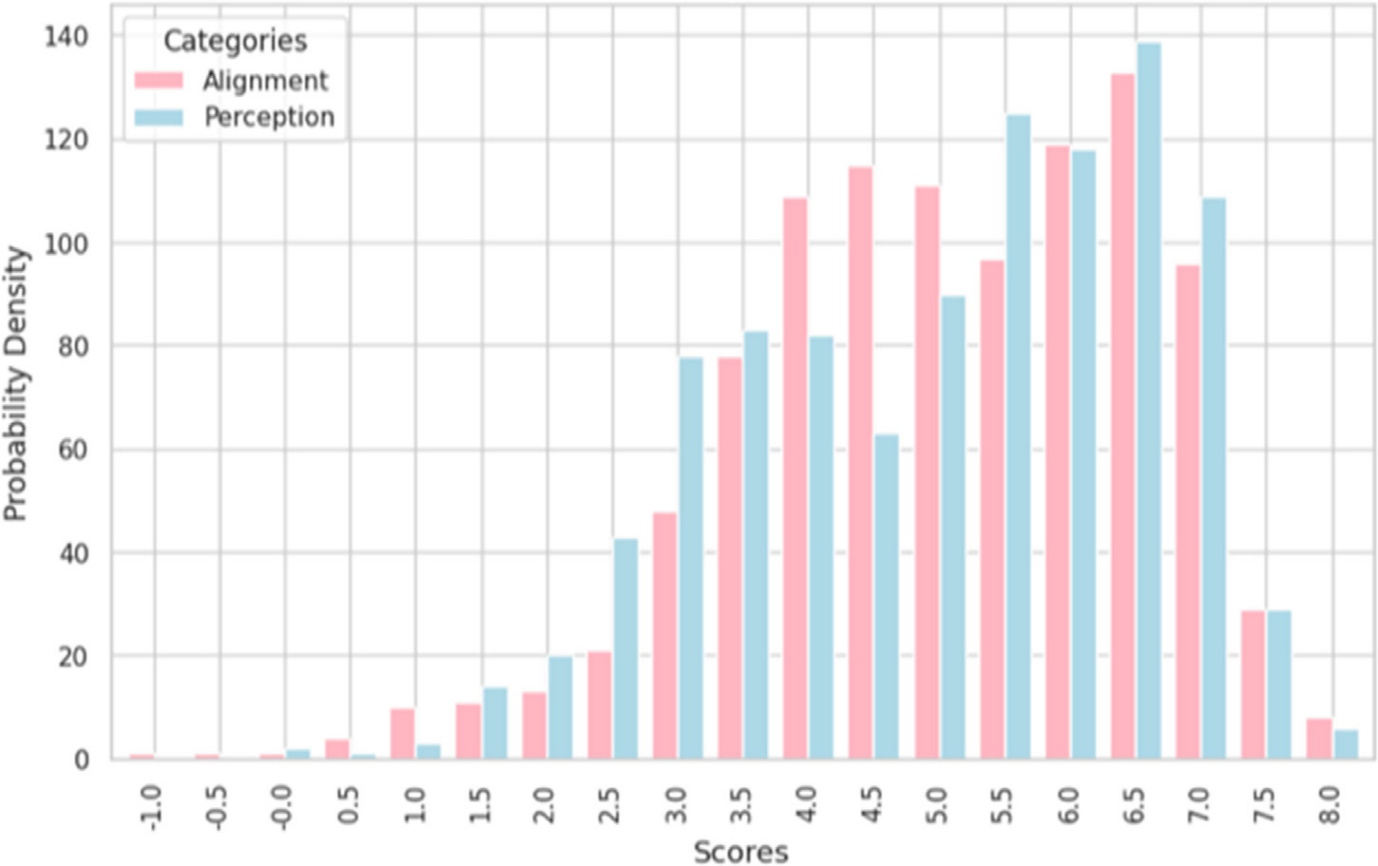
Distribution of adjusted MOS Scores.

**Fig. 4:**
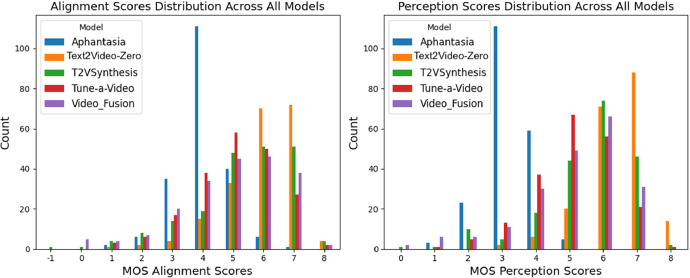
MOS score distributions across different models.

Fig. 3 shows the distribution of adjusted MOS scores for alignment and perception for all 1005 videos while Fig. 4 shows the distributions on a per-model basis. Table 1 shows the mean and standard deviations of MOS scores for alignment and perception for videos generated by each of the 5 models.

**Table 1:** Human evaluation MOS scores for five text-to-video models.

Model	Alignment		Perception	
	Mean	Std.Dev.	Mean	Std.Dev.
Aphantasia	4.016	0.841	3.221	0.692
Text2Video-Zero	5.985	1.139	6.393	0.886
T2VSynthesis	5.333	1.622	5.485	1.366
Tune-a-Video	5.053	1.340	5.070	1.196
Video Fusion	4.995	1.686	5.139	1.507

## Limitations

None.

## Ethics Statement

This research was carried out in accordance with the Declaration of Helsinki, and includes ethical approval from the School of Computing Research Ethics Committee 2023-01-20. Informed consent was obtained from human participants who provided video annotations.

## CRediT authorship contribution statement

**Iya Chivileva:** Conceptualization, Methodology, Software, Validation, Formal analysis, Writing – original draft, Visualization. **Philip Lynch:** Conceptualization, Methodology, Software, Validation, Formal analysis, Writing – original draft, Visualization. **Tomás E. Ward:** Methodology, Validation, Data curation, Writing – review & editing, Supervision. **Alan F. Smeaton:** Conceptualization, Methodology, Validation, Data curation, Writing – review & editing, Supervision.

## Data Availability

Text prompts and videos generated using 5 popular Text-to-Video models plus quality metrics including user quality assessments (Original data) (Figshare).
